# Factors associated with musculoskeletal symptoms in professionals working in sitting position

**DOI:** 10.11606/s1518-8787.2021055002617

**Published:** 2021-03-29

**Authors:** Anália Rosário Lopes, Celita Salmaso Trelha, Maria Lúcia do Carmo Cruz Robazzi, Roberta Alvarenga Reis, Maria José Bistafa Pereira, Claudia Benedita dos Santos

**Affiliations:** I Universidade Federal da Integração Latino-Americana Instituto Latino-Americano de Ciências da Vida e da Natureza Foz do IguaçuPR Brasil Universidade Federal da Integração Latino-Americana. Instituto Latino-Americano de Ciências da Vida e da Natureza. Curso de Medicina. Foz do Iguaçu, PR, Brasil; II Universidade Estadual de Londrina Departamento de Fisioterapia LondrinaPR Brasil Universidade Estadual de Londrina. Departamento de Fisioterapia. Londrina, PR, Brasil; III Universidade de São Paulo Escola de Enfermagem de Ribeirão Preto Departamento de Enfermagem Geral e Especializada Ribeirão PretoSP Brasil Universidade de São Paulo. Escola de Enfermagem de Ribeirão Preto. Departamento de Enfermagem Geral e Especializada. Ribeirão Preto, SP, Brasil; IV Universidade Federal do Rio Grande do Sul Faculdade de Odontologia Departamento de Odontologia Preventiva e Social Porto AlegreRS Brasil Universidade Federal do Rio Grande do Sul. Faculdade de Odontologia. Departamento de Odontologia Preventiva e Social. Porto Alegre, RS, Brasil; V Universidade de Ribeirão Preto Programa de Mestrado Profissional Educação em Saúde Ribeirão PretoSP Brasil Universidade de Ribeirão Preto Campus Ribeirão. Programa de Mestrado Profissional Educação em Saúde. Ribeirão Preto, SP, Brasil; VI Universidade de São Paulo Escola de Enfermagem de Ribeirão Preto Departamento de Enfermagem Materno Infantil e Saúde Pública Ribeirão PretoSP Brasil Universidade de São Paulo. Escola de Enfermagem de Ribeirão Preto. Departamento de Enfermagem Materno Infantil e Saúde Pública. Ribeirão Preto, SP, Brasil

**Keywords:** Occupational Health, Measures of Association, Exposure, Risk or Outcome, Cumulative Trauma Disorders, Posture, Sedentary Lifestyle

## Abstract

**OBJECTIVE:**

To estimate the prevalence of musculoskeletal symptoms and analyze their associated factors in professionals from administrative sectors working predominantly in sitting position.

**METHODS:**

This is a cross-sectional study with data obtained from 451 workers from a federal public institution in Southern Brazil. The dependent variable was the number of musculoskeletal symptoms in the prior 12 months, measured using the Nordic Musculoskeletal Questionnaire. In the analyses, 19 independent variables were investigated, divided into four categories: sociodemographic, behavioral, occupational and health characteristics. Univariate analysis and multiple Poisson regression with robust variance were performed. The independent variables were inserted into blocks with stepwise backward criterion, considering the value for Wald statistics equal to 0.20. The effect measures were expressed in a relative increase (RI) in the mean value, and the data were analyzed for a 5% significance level.

**RESULTS:**

The estimated prevalence of musculoskeletal symptoms in the prior 12 months was 90% (confidence interval – 95%CI 87–93). In the final model of regression analysis, the variables female gender (RI = 14.75%), low (RI = 100.02%) and moderate (RI = 64.06%) work ability index, use of medications (RI = 48.06%) and waist circumference at risk (RI = 15.59%) had a significant association with the increase in the mean number of symptoms; schooling with technical education acted as a protective factor, reducing the mean by 36.46%.

**CONCLUSIONS:**

The high prevalence of musculoskeletal symptoms found and the associated factors indicate the need to propose specific actions and care for this population, such as immediate treatment of symptoms and changes in the organization and work environment, to achieve balance and harmony in the demands of prolonged sitting work and avoid its impact effect of this condition on public health.

## INTRODUCTION

Workers’ health is an area of public health that has as object of study and intervention the relations between work and health, in which social, political and technical dimensions are inseparable. This area was not linearly incorporated within the scope of the Unified Health System (SUS), thus requiring the overcoming of obstacles, multiprofessional training, efforts in the articulation between its instances and interinstitutional support – which have obtained advancements, but still present many challenges^1^.

The effective prevention of workers’ health problems demands transformations combined with social support and protection. Work-related musculoskeletal disorders (WRMD) are high prevalent, and the recognition of the factors associated with them and their determinants is essential^2^.

In addition, there is a growing interest in knowing the effects that sitting for a long time causes in the health of individuals^3^. This position is increasingly frequent in work positions, which has encouraged researchers^7^ to quantitatively evaluate the acceptability, viability and perceptions of office workers, of the use of sit-stand workstations, which allow the alternation between sitting and standing during working hours.

Moreover, there is greater awareness and concern in distinguishing the effects of physical inactivity and sedentary lifestyle, since an individual can be classified as active according to the definition standardized by the World Health Organization (WHO)^8^ and, at the same time, have a sedentary lifestyle, which is the case of those sitting for a long time^9^.

Since the WRMD are problems of epidemic dimension^10^ in several professional categories, with great social, economic and health impact, especially in administrative or office activities, in which workers remain seated for long periods, in conditions that require further studies and investigations, it is observed the relevance of identifying the main factors associated with the musculoskeletal symptoms (MS) of these workers, enabling a more efficient preventive and interventional approach. Thus, our study aims at estimating the prevalence of MS and analyzing their associated factors in professionals from administrative sectors that work predominantly in sitting position.

## METHODS

This is a cross-sectional study, conducted in an organ of the federal public administration of two municipalities in the Southern Brazil, with adult workers from administrative sectors that work predominantly in the sitting position.

Those eligible for participation were individuals: a) aged between 18 and 59 years; b) who had attended school at least up to the fifth grade; c) who have worked at the institution for at least six months; and d) who have been working in administrative sectors in the sitting position for at least six months.

A predominantly sitting position was considered during the working hours, the one in which the professionals remain more than 50% of their daily workload in this position – a condition self-reported by the participant. Self-report on sitting time is used in many studies and considered reliable^5,6^.

The following workers were excluded: a) workers on work leave or vacation up to one month before or during data collection; b) pregnant women; c) workers that had MS as a result of neurological, congenital, rheumatic or neoplastic diseases; d) workers presenting deformity that impaired physical tests; e) workers with vocal and/or auditory impairment; and f) workers that had suffered fall or trauma in the previous three months.

For the sample calculation, a mean prevalence of WRMD equal to 60%, a significance level of α = 0.05, estimation error of d = 0.05 and an increase of 15% for possible losses were considered, reaching a sample of 434 workers. The sampling was done for convenience with the workers of the two previously selected municipalities.

### Data Collection Instruments

To collect information on the independent variables, a structured questionnaire was developed to obtain sociodemographic data, behavioral variables, work factors and health characteristics with possible associations with WRMD. This questionnaire had the validity of its content analyzed by a team of seven physician before the beginning of the study, obtaining a 0.98 content validity index.

Thus, the questionnaire consisted of 19 independent variables, separated into four classes: 1) sociodemographic (gender, age and schooling); 2) behavioral (physical activity, smoking, time in sitting position, in addition to work and computer use after work); 3) occupational (overtime, daily time sitting at work and working time predominantly in sitting position – considering previous occupations, frequency of computer use, ergonomic conditions of the job position and work ability index – WAI); and 4) health (use of medication for musculoskeletal pain/discomfort in the previous 12 months, waist circumference, flexibility of the posterior muscle chain, muscle strength of the lower part of the abdominals, shortening of hip flexors and resistance of the abdominal muscles).

The dependent variable was the MS prevalence in the prior 12 months, investigated with the Nordic Musculoskeletal Questionnaire (NMQ), in its general version, already validated in Brazil^13^. The interpretation of this outcome was performed numerically, preserving the reports of the workers, who could mention none (zero) or even nine MS.

For the identification related to the practice of physical activity, the WHO classification was used, which recommends for adults 150 minutes of moderate aerobic physical activity per week, or at least 75 minutes in vigorous intensity, and also accepts an equivalent combination of moderate and vigorous activities^8^.

The evaluation of the ergonomic conditions of computer workers was conducted with Coutos’s checklist, version 2014^14^. The WAI was used in a translated version adapted for Brazil to evaluate work capacity^15^.

In our study, we chose to use the terminology “waist circumference” (WC) based on a systematic review^16^, the midpoint between the last rib and the iliac crest as an anatomical site of measurement and cutoff points according to the International Diabetes Federation^17^ – the standard most used internationally and adopted by the Brazilian Society of Cardiology.

The strength of the lower abdominal muscles was identified according to the procedure suggested by Kendall et al.^18^ Thomas’s test was used to evaluate the shortening of the hip flexor muscles, according to Magee^19^. The flexibility of the muscles of the posterior region of the trunk and lower limbs, also called the posterior muscle chain, was measured by the sit-and-reach test with Wells’ bench, because it is a method widely used in studies^20^.

The endurance of the abdominal muscles was evaluated with the one-minute abdominal test, which consists of performing the maximum number of abdominals possible in this time interval. The normative values for this test consider gender and age and classify endurance as excellent, above average, average, below average or weak^21^.

### Data Collection and Analysis

Data collection was performed by a team of five examiners, who received a 20-hour training on aspects related to interview techniques, methods and procedures of tests and instruments, aiming at standardization and calibration. A group was created in a communication application on the phone to resolve immediate doubts, in addition to weekly face-to-face meetings. The interviews and physical tests were conducted in a private room in the workplace, – due to the use of Wells mattress and bench and to preserve the participant’s privacy – with an average duration of 35 minutes.

### Statistical Analysis

The results were showed with a descriptive and analytical approach. In the descriptive approach, the absolute and relative frequency distribution and measures of central tendency to describe quantitative variables are presented. Regarding the analytical approach, a 95% confidence interval was obtained to estimate the MS prevalence, and univariate analyses were performed in the comparison of all independent and dependent variables. In the definition of the comparison groups according to the categories of the variable or in the categorization of numerical variables, for large samples (n > 30) the Student’s *t-*test (for two independent groups) and Anova (for three or more independent groups) were used. In all cases, intervals with 95% confidence were obtained for the means. The Pearson’s linear correlation test was used for the numerical variables, without categorization.

In the small samples and in the case of unsatisfied normality, verified by the Shapiro-Wilk test, the Kruskal-Wallis nonparametric test (for three or more independent groups) was used. The homoscedasticity among groups was verified by the Levene test (parametric) or the Fligner-Killer test (nonparametric). The Poisson regression analysis with robust variance was chosen, since it is a reference for the analysis of counting data and because the odds ratio tends to overestimate the prevalence ratio when the outcome is common or high^22^.

We used a model, in which the independent variables were inserted into blocks in the following order: sociodemographic, behavioral, occupational and health data. Variables with significance lower than 0.20 (p < 0.20) were included in univariate analyses. The selection of variables in the model was performed with the stepwise backward criterion, also considering a value equal to 0.20 for Wald statistics in the maintenance of variables during the level-adjusted analysis to control potential confounding factors. The effect measures were expressed in a relative increase (RI) in the mean.

The final analyses were considered at a 5% significance level (α = 0.05). The tests were performed in the statistical programs R version 3.4.2 and Statistical Package for the Social Sciences (SPSS) version 22.0. The research was approved on November 21, 2017 by the Research Ethics Committee involving Human Beings of the Escola de Enfermagem Ribeirão Preto of the Universidade de São Paulo under the CAAE protocol 74543517.8.0000.5393, and all participants signed an informed consent form.

## RESULTS

The study was conducted with 451 workers, with a mean age of 44.4 years, mostly women (54.5%), who attended undergraduate or graduate course (81.2%), non-smokers (84.3%), and practicing physical activity regularly (53.9%). They remained seated for 6.51 hours on average at work and another 3.12 hours during leisure/rest time. Table 1 shows other behavioral, occupational and health characteristics and Table 2, the numerical variables. There was no statistical significance in any correlation analysis of numerical variables with the number of MS in the previous 12 months reported by the workers (Table 2).

**Table 1:** Distribution of participants according to sociodemographic, behavioral, occupational and health characteristics (n = 451) in municipalities in Southern Brazil.

		Frequency (n)	Percentage (%)
Gender	Female	246	54.5
	Male	205	45.5
Education level	High school	62	13.7
	Technical Education	23	5.1
	Higher education	260	57.6
	Graduate studies	106	23.6
Smoking	Daily smoker	17	3.8
	Occasional smoker	9	2.0
	Former smoker	45	10.0
	Non-smoker	380	84.3
Practice of physical activity	Yes	243	53.9
	No	208	46.1
Computer use outside of work	Yes	179	39.7
	No	272	60.3
Overtime	Yes	34	7.5
	No	417	92.5
Frequency of computer use at work	Less than 50% of the time	13	2.9
	Between 50% and 70% of the time	35	7.8
	Less than 50% of the time	403	89.4
WAI	Low	6	1.3
	Moderate	79	17.5
	Good	204	45.2
	Optimal	162	35.9
Use of medication in the last 12 months	Yes	244	54.1
	No	207	45.9
Waist circumference	Lower risk	202	44.8
	Increased risk	147	32.6
	Substantially increased risk	102	22.6
Flexibility (Wells Bank)	Excellent	41	9.1
	Above average	63	14.0
	Average	69	15.3
	Below average	81	18.0
	Very poor	197	43.7
Muscle strength of the lower abdominals	Regular	170	37.7
	Good	235	52.1
	Normal	46	10.2
Resistance of the abdominals	Excellent	94	20.8
	Above average	81	18.0
	Average	74	16.4
	Below average	66	14.6
	Weak	136	30.2
Shortening of hip flexors	Yes	80	17.7
	No	371	82.3

WAI: work ability index.

**Table 2:** Descriptive data and correlation analyses of numerical variables with the amount of musculoskeletal symptoms in the prior 12 months (n = 451) in municipalities in Southern Brazil.

	Age (years)	Time sitting not at work (hours/day)	Time sitting at work (hours/day)	Sitting time at work (in years)	Couto’s checklist final average (in %)
Descriptive data: mean value and standard deviation (SD)	44.4 (DP = 10.6)	3.12 (DP = 1.55)	6.51 (DP = 0.96)	20.29 (DP = 10.88)	88.9% (DP = 4.37)
Amount of MS in the last 12 months					
Correlation coefficient^a^	0.083	0.082	0.033	0.079	0.068
Bilateral significance	0.077	0.082	0.489	0.093	0.152

MS: musculoskeletal symptoms.

^a^ Spearman’s correlation test.

The estimated prevalence of MS in the prior 12 months was 90% (95%CI 87–93), with an average of 3.34 symptoms per participant. The analysis of MS regarding the sociodemographic variables showed a higher frequency among women, with an average of 3.68 MS in the prior 12 months, whereas men obtained an average of 2.94, with p < 0.001. Regarding education, those with technical education had the lowest MS median compared to participants with other levels of education, with p = 0.027 (Table 3).

Regarding behavioral variables, physically inactive individuals had higher MS mean (*x¯* = 3.62) than those active (*x¯* = 3.11), with p = 0.014. In the analysis of smoking and computer use outside work, no significant differences were observed among the groups (Table 3).

Regarding the relationship of MS with occupational variables, the analyses showed that workers with low WAI also had more MS, with a median of 5.5, whereas those with excellent WAI had a median of 2, with p < 0.001 (Table 4).

**Table 4:** Sociodemographic and behavioral data regarding musculoskeletal symptoms in the prior 12 months (n = 451) in municipalities in Southern Brazil.

		Average	Standard deviation	95%CI	Median	Minimum	Maximum	p
Overtime	Yes	3.15	1.893	2.49–3.81	3.00	0	7	0.587
	No	3.36	2.214	3.15–3.57	3.00	0	9	
Student’s *t*-test								
Frequency of computer use at work	Less than 50% of the time	-	-	-	2.00	1	7	0.614
	Between 50% and 70% of the time	-	-	-	3.00	0	8	
	Less than 50% of the time	-	-	-	3.00	0	9	
Kruskal-Wallis test								
WAI	Low	-	-	-	5.50	2	9	**< 0.001**
	Moderate	-	-	-	5.00	0	9	
	Good	-	-	-	3.50	0	9	
	Optimal	-	-	-	2.00	0	8	
Kruskal-Wallis test								
Use of medication in the last 12 months	Yes	4.10	2.031	3.85–4.36	4.00	0	9	**< 0.001**
	No	2.45	2.033	2.17–2.73	2.00	0	9	
Student’s *t*-test								
Waist circumference	Lower risk	3.08	2.098	2.79–3.37	3.00	0	9	**< 0.001**
	Increased risk	3.12	2.225	2.75–3.48	3.00	0	9	
	SIR	4.20	2.125	3.78–4.61	4.00	0	9	
Anova test								
Flexibility (Wells Bank)	Excellent	-	-	-	3.00	0	7	0,602
	Above average	-	-	-	3.00	0	8	
	Average	-	-	-	3.00	0	9	
	Below average	-	-	-	3.00	0	9	
	Very poor	-	-	-	3.00	0	9	
Kruskal-Wallis test								
Muscle strength of the lower abdominals	Regular	3.61	2.182	3.28–3.94	3.50	0	9	0.134
	Good	3.17	2.248	2.88–3.45	3.00	0	9	
	Normal	3.28	1.846	2.73–3.83	3.00	0	8	
Anova test								
Resistance of the abdominals	Excellent	2.85	2.190	2.40–3.30	2.50	0	9	0.072
	Above average	3.40	2.017	2.95–3.84	3.00	0	9	
	Average	3.46	2.121	2.97–3.95	3.00	0	8	
	Below average	3.17	2.351	2.59–3.74	3.00	0	9	
	Weak	3.68	2.211	3.30–4.05	3.50	0	9	
Anova test								
Shortening of hip flexors	Yes	3.63	2.420	3.09–4.16	3.00	0	9	0.245
	No	3.28	2.137	3.06–3.50	3.00	0	9	
Student’s *t*-test								

MS: musculoskeletal symptoms; WAI: work ability index; SIR: substantially increased risk; 95%CI: interval with 95% confidence.

Values with statistical significance are shown in bold.

The univariate analyses of the health variables regarding the presence of MS in the prior 12 months showed that people that used pain medications in the previous year had higher mean MS (*x¯* = 4.10) than those that did not use (*x¯* = 2.45), with p < 0.001 (Table 4).

Workers classified by measuring waist circumference, in the category of substantially increased risk for metabolic diseases and other complications had a higher MS mean (4.20) than those who at lower risk (3.08), with p <0.001. In the flexibility analysis, no statistically significant difference was observed among the groups (p = 0.602), and all categories of classifications had a median equal to 3 (Table 4).

Evaluating the relation with the muscle strength of the lower abdominals, those with regular strength recorded an average MS of 3.61 and 95%CI 3.28–3.94. In the study of abdominal muscle resistance, evaluated in the one-minute test, no significant difference was found between the groups, with the average of workers with weak muscle resistance of 3.68 MS. With a very similar value, those with shortening of the hip flexors had an average MS *x¯* of = 3.63 and 95%CI 3.09–4.16, with no significant difference between the groups (Table 4).

Based on the results of the univariate analyses, twelve variables were selected for the regression model: age; gender; schooling; practice of physical activity; time sitting outside of work; time in work predominantly in sitting position; ergonomic conditions of the workstation; WAI; use of medicines in the prior 12 months; waist circumference; muscle strength of the lower abdominals; and abdominal resistance.

The results of the multiple regression analysis, after all the steps by blocks (sociodemographic, behavioral, occupational and health variables), showed five factors significantly associated with the MS mean. Table 5 presents all the variables that remained in the model until the end of the analysis and their respective estimated values of RI in the mean, 95%CI and statistical significance (p-value).

**Table 5:** Poisson regression model with robust variance in (n = 451) municipalities in Southern Brazil.

Parameters	Estimate	Standard error	z-value	p-value	RI	95%CI	
*(Intercept)*	0.6763	0.1090	6.2061	0.0000	1.9667		
Female	0.1376	0.0626	2.1967	**0.0280**	1.1475	1.0149	1.2974
Education: technical education	-0.4535	0.1788	-2.5361	**0.0112**	0.6354	0.4475	0.9021
Education: technical education	-0.1134	0.0851	-1.3323	0.1828	0.8928	0.7556	1.0549
Education: graduate	-0.0573	0.0999	-0.5739	0.5660	0.9443	0.7764	1.1485
Low WAI	0.6933	0.2050	3.3817	**0.0007**	2.0002	1.3384	2.9894
Moderate WAI	0.4950	0.0868	5.7035	**0.0000**	1.6406	1.3839	1.9448
Use of medicines in the past 12 months	0.3924	0.0643	6.1008	**0.0000**	1.4806	1.3052	1.6795
Increased risk	-0.0421	0.0702	-0.5996	0.5488	0.9588	0.8355	1.1002
WC in substantially increased risk	0.1449	0.0738	1.9630	**0.0496**	1.1559	1.0002	1.3358

RI: relative increase in mean; 95%CI: interval with 95% confidence; WAI: work ability index; WC: waist circumference.

Values with statistical significance are shown in bold.

Women had an RI in the MS mean of 14.75% (p = 0.0280) compared to men. In the analysis of schooling, workers with technical education showed a reduction of 36.46% (p = 0.0112) in the MS mean compared to those with high school education, showing that this is a protective factor (Table 5).

Workers with low and moderate WAI presented RI of 100.02% (p = 0.0007) and 64.06% (p < 0.0001) in the mean number of symptoms, respectively, compared to those with optimal WAI. On the other hand, participants that used musculoskeletal pain medications in the last 12 months had RI 48.06% on average (p < 0.0001) when compared with those that did not use medications (Table 5).

Finally, as of waist circumference, participants at substantially increased risk for metabolic syndrome and other complications presented RI in the mean MS of 15.59% (p = 0.0496) compared to workers at lower risk (Table 5).

## DISCUSSION

The estimated prevalence of MS in the prior 12 months was high (90%). Some studies have found a high prevalence of musculoskeletal disorders among individuals that work predominantly seated, such as office (88.4%)^10^ and computer workers (76%)^11^.

Pain was significantly more frequent in women when compared with men, with RI of 14.75% in the mean number of symptoms. This data is regularly reported by authors that study MS in workers. In a study by Scopel et al.^12^, men had a lower prevalence of cases suggestive of WRMD, with an estimated prevalence ratio of 0.62 (95%CI 0.47–0.81).

Among the office workers that use a computer, the women presented more MS in all the anatomical regions evaluated, and in the analysis of multivariate association obtained a odds ratio equal to 2.4 (p = 0.03) for the cervical region and equal to 2.8 (p = 0.01) for upper limbs (arm, elbow, forearm, wrist or hand)^11^. Some possible explanations for these findings would be the lower muscle mass, in absolute and relative terms, in the physical composition of the woman^19^; changes in the hormonal system; and the double working day, since women are often responsible for family care.

In the analysis of schooling, those who had a technical course presented fewer symptoms, which works as a protective factor. There seems to be no established pattern for this variable. Some studies have shown no association between schooling and MS^23,24^, whereas others have found an association of MS with a lower level of studies^12,25^.

The workers classified with low WAI presented an RI in the significant MS mean compared to those with optimal WAI. Iunes et al.^26^ showed an association between WAI and the presence of symptoms in all regions described in the NMQ.

Walsh et al.^27^ observed that, among workers with poor/low WAI, 87% had pain intensity between 7 and 10, whereas 73% of those with excellent WAI scored from 0 to 2. In the study by Martinez and Latorre^28^, all health dimensions analyzed, including pain assessment, were associated with work ability, which is much better the higher the quality of health.

This relationship seems to occur in both directions, that is, the worker that has a high number of MS tends to have a low WAI, and the one with a high WAI has a lower tendency to present work-related MS. Maintaining work capacity is a challenge for health services and WAI and, together with other assessments, provide professionals and managers with essential tools and data to monitor workers’ health, enabling prevention and health promotion measures in the workplace.

By analyzing the health variables, we observed a statistically significant difference between those that used medication for musculoskeletal pain in the previous year and those that did not. In the regression analysis, a significant association was observed, expressing a 48.06% RI in the mean of symptoms. This data can be easily understood, since the person in pain is more likely to use medications. In a study by Souza et al.^29^, 64.51% of the workers of a public hospital used pain medication in the week before to the study.

The association of waist circumference of workers classified as at substantially increased risk (indicator of central or abdominal obesity) with MS mean remained statistically significant in the univariate analysis until the final Poisson regression model, with a 15.59% RI. Magnago et al.^25^ found that almost half of the nursing workers of a large university hospital in Rio Grande do Sul were above the ideal weight, and this data was significantly associated with reports of joint and lumbar spine pain.

For a possible understanding of this association, it is worth mentioning the effect of joint overload that occurs in overweight and obese people. Stress and excessive pressure cause wear on the structures and tissues involved (cartilage, ligaments, tendons, muscles, among others), predisposing to degeneration and pain^30^.

As limitations of our study we cite the presentation and analysis of MS data from the numerical perspective, which considers the influence of each reported symptom and maintains coherence with the mathematical model of Poisson regression. Another observation is the clinical presentation, which may be more relevant to the health professional, since in preventive and/or rehabilitative interventions it is important to identify the associated factors capable of increasing the MS number in workers, without being restricted itself to each body region of the NMQ.

In addition to the typical limitations of cross-sectional studies, such as the impossibility of establishing causality, it is worth mentioning the question of self-report of sitting time, although the literature already demonstrates some reliability. There was no bias of the healthy participant, given the high MS prevalence; however, the bias of the healthy worker should be considered, because of the exclusion of workers in work leave due to illness.

Our study contributes to the advancement of knowledge about the health of workers that, regardless of whether they practice physical activity or not, can be classified as a sedentary behavior/lifestyle, since the nature of their professional occupation requires sitting position for long periods, on daily basis^9^, so that they are subjected to this risk factor, considered a distinct construct of physical inactivity and growing interest in public health.

Therefore, the variables female gender, low and moderate WAI, use of medications in the prior 12 months and waist circumference above the recommended were statistically associated with increased MS, with the WAI variable being the one with the highest power of association. There was also a high MS prevalence in these workers. This said, interventions to reduce MS will positively affect work ability, which, in turn, tends to decrease the presence of symptoms.

Therefore, an immediate measure to be adopted is the rehabilitation of workers with MS. Initial symptoms, if neglected, can evolve to disabling disorders, making it necessary to use government removals and aid – which increases a major public health problem in Brazil, with repercussions on social and economic dimensions.

Moreover, some specific actions are proposed: physical adaptations in the workplace and adjustments/changes in the organization of activities, so that workers can stand for a few minutes every hour and have higher energy expenditure; guidance and clarification on risk behaviors and healthy habits in the work environment with specialized health professionals; and performing specific exercises for the musculoskeletal system, which stimulate blood circulation and develop individual physical characteristics proven protective for those that sit for long periods.

**Table 3:** Sociodemographic and behavioral data regarding musculoskeletal symptoms in the prior 12 months (n = 451) in municipalities in Southern Brazil.

		Average	Standard deviation	95%CI	Median	Minimum	Maximum	p
Gender	Female	3.68	2.168	3.41–3.96	4.00	0	9	**< 0.001**
	Male	2.94	2.154	2.64–3.23	3.00	0	9	
Student’s t-test								
Education level	High school	-	-	-	3.00	0	9	**0.027**
	Technical Education	-	-	-	2.00	0	6	
	Higher education	-	-	-	3.00	0	9	
	Graduate studies	-	-	-	3.00	0	9	
Kruskal-Wallis test								
Smoking	Daily smoker	-	-	-	5.00	0	8	0.397
	Occasional smoker	-	-	-	4.00	1	6	
	Former smoker	-	-	-	3.00	0	7	
	Non-smoker	-	-	-	3.00	0	9	
Kruskal-Wallis test								
Practice of physical activity	Yes	3.11	2.078	2.84–3.37	3.00	0	9	**0.014**
	No	3.62	2.289	3.31–3.93	4.00	0	9	
Student’s *t*-test								
Using the computer outside of work	Yes	3.23	2,173	2,91–3,56	3,00	0	9	0,392
	No	3.42	2,204	3,15–3,68	3,00	0	9	
Student’s *t*-test								

95%CI: 95% confidence interval.

Values with statistical significance are shown in bold.
