# Supplementary material for: Factors associated with musculoskeletal symptoms in professionals working in sitting position
Source: Rev Saude Publica. 2021 Mar 29;55:2. doi: 10.11606/s1518-8787.2021055002617 (PMC8009318; doi:10.11606/s1518-8787.2021055002617)
Supplement: Translation

# Translation: Fatores associados a sintomas osteomusculares em profissionais que trabalham sentados

**Authors:** Anália Rosário Lopes, Celita Salmaso Trelha, Maria Lúcia do Carmo Cruz Robazzi, Roberta Alvarenga Reis, Maria José Bistafa Pereira, Claudia Benedita dos Santos

**Affiliations:** I Brasil Universidade Federal da Integração Latino-Americana. Instituto Latino-Americano de Ciências da Vida e da Natureza. Curso de Medicina. Foz do Iguaçu, PR, Brasil; II Brasil Universidade Estadual de Londrina. Departamento de Fisioterapia. Londrina, PR, Brasil; III Brasil Universidade de São Paulo. Escola de Enfermagem de Ribeirão Preto. Departamento de Enfermagem Geral e Especializada. Ribeirão Preto, SP, Brasil; IV Brasil Universidade Federal do Rio Grande do Sul. Faculdade de Odontologia. Departamento de Odontologia Preventiva e Social. Porto Alegre, RS, Brasil; V Brasil Universidade de Ribeirão Preto Campus Ribeirão. Programa de Mestrado Profissional Educação em Saúde. Ribeirão Preto, SP, Brasil; VI Brasil Universidade de São Paulo. Escola de Enfermagem de Ribeirão Preto. Departamento de Enfermagem Materno Infantil e Saúde Pública. Ribeirão Preto, SP, Brasil

**Keywords:** Saúde do Trabalhador, Medidas de Associação, Exposição, Risco ou Desfecho, Transtornos Traumáticos Cumulativos, Postura, Estilo de Vida Sedentário

## Abstract

**OBJETIVOS:**

Estimar a prevalência de sintomas osteomusculares e analisar os fatores a eles associados em profissionais de setores administrativos que trabalham predominantemente na postura sentada.

**MÉTODOS:**

Trata-se de estudo transversal com dados obtidos de 451 trabalhadores de instituição pública federal na região Sul do país. A variável dependente foi o número de sintomas osteomusculares nos últimos 12 meses, aferido utilizando-se o Questionário Nórdico de Sintomas Osteomusculares. Foram investigadas 19 variáveis independentes, divididas em quatro categorias: características sociodemográficas, comportamentais, ocupacionais e de saúde. Foi realizada análise univariada e, na sequência, regressão múltipla de Poisson com variância robusta. As variáveis independentes foram inseridas em blocos com critério *backward stepwise*, considerando o valor para estatística de Wald igual a 0,20. As medidas de efeito foram expressas em aumento relativo (AR) no valor médio, sendo os dados analisados para um nível de significância de 5%.

**RESULTADOS:**

A prevalência estimada de sintomas osteomusculares nos últimos 12 meses foi de 90% (intervalo de confiança – IC95% 87–93). No modelo final da análise de regressão, as variáveis sexo feminino (AR = 14,75%), índice de capacidade para o trabalho baixo (AR = 100,02%) e moderado (AR = 64,06%), uso de medicamentos (AR = 48,06%) e circunferência da cintura em risco (AR = 15,59%) tiveram associação significativa com o aumento da média de sintomas; já a escolaridade com ensino técnico atuou como fator de proteção, reduzindo a média em 36,46%.

**CONCLUSÕES:**

A alta prevalência de sintomas osteomusculares encontrada e os fatores associados indicam a necessidade de propor ações e cuidados específicos para essa população, como tratamento imediato dos sintomas e mudanças na organização e no ambiente laboral, a fim de alcançar equilíbrio e harmonia nas exigências do trabalho sentado prolongado e evitar o impacto dessa condição na saúde pública.

## INTRODUÇÃO

A saúde do trabalhador é uma área da saúde pública que tem como objeto de estudo e intervenção as relações entre trabalho e saúde, com dimensões sociais, políticas e técnicas indissociáveis. No âmbito do Sistema Único de Saúde (SUS), a incorporação dessa área não ocorreu de modo linear, mas demandou a superação de obstáculos, formação multiprofissional, esforços na articulação entre suas instâncias e apoios interinstitucionais – que obtiveram avanços, porém ainda apresentam muitos desafios^1^.

A efetiva prevenção dos agravos à saúde do trabalhador demanda transformações aliadas ao apoio e à proteção social. Destaca-se a alta prevalência de distúrbios osteomusculares relacionados ao trabalho (DORT), sendo premente o reconhecimento dos fatores a eles associados e de seus determinantes^2^.

Além disso, existe um crescente interesse em conhecer os efeitos que a postura sentada por tempo prolongado causa à saúde dos indivíduos^3^. Essa posição é cada vez mais frequente nos postos de trabalho, o que vem estimulando pesquisadores^7^ a avaliar, quantitativamente, a aceitabilidade, a viabilidade e as percepções, pelos trabalhadores de escritório, do uso de estações de trabalho sit-stand, que permitem a alternância entre ficar sentado e em pé durante o expediente.

Ademais, há maior conscientização e preocupação em distinguir os efeitos da inatividade física e do estilo de vida sedentário, pois um indivíduo pode ser classificado como ativo segundo a definição padronizada pela Organização Mundial da Saúde (OMS)^8^ e, ao mesmo tempo, ter um estilo de vida sedentário, que é o caso de quem permanece muito tempo sentado por dia^9^.

Visto que os DORT constituem problema de dimensão epidêmica^10^ em diversas categorias profissionais, com grande impacto social, econômico e de saúde, principalmente nas atividades administrativas ou de escritório, nas quais os trabalhadores permanecem sentados por longos períodos, em condições que requerem mais estudos e investigações, constata-se a relevância de identificar os principais fatores associados aos sintomas osteomusculares (SO) desses trabalhadores, possibilitando uma abordagem preventiva e intervencionista mais eficiente. Dessa forma, esta pesquisa tem como objetivos estimar a prevalência de SO e analisar os fatores a eles associados em profissionais de setores administrativos que trabalham predominantemente na postura sentada.

## MÉTODOS

Trata-se de estudo transversal, realizado em órgão da administração pública federal de dois municípios da região Sul do Brasil, com trabalhadores adultos de setores administrativos que atuam, predominantemente, na postura sentada.

Os elegíveis para participação foram pessoas: a) com idade entre 18 e 59 anos, inclusive; b) que tivessem cursado, no mínimo, até o quinto ano do ensino fundamental; c) que trabalhassem na instituição há no mínimo seis meses; e d) que atuassem em setores administrativos na postura sentada há pelo menos seis meses.

Foi considerada postura predominantemente sentada durante o expediente, aquela em que o profissional permanece mais que 50% de sua carga horária diária de trabalho nessa postura – condição autorrelatada pelo participante. O autorrelato sobre o tempo sentado é utilizado em muitos estudos e tem-se mostrado confiável^5,6^.

Foram excluídos do estudo aqueles: a) em afastamento do trabalho, licença ou férias até um mês antes ou durante a coleta de dados; b) gestantes; c) que possuíssem SO em consequência de doenças neurológicas, congênitas, reumáticas ou neoplásicas; d) que apresentassem deformidade limitante para a realização dos testes físicos; e) com deficiência vocal e/ou auditiva; e f) que tivessem sofrido queda ou trauma nos últimos três meses.

Para o cálculo amostral foi considerada prevalência média de DORT igual a 60%, nível de significância α = 0,05, erro de estimativa d = 0,05 e acréscimo de 15% para possíveis perdas, alcançando-se uma amostra de 434 trabalhadores. A amostragem foi feita por conveniência com os trabalhadores dos dois municípios previamente selecionados.

### Instrumentos de Coleta de Dados

Para coleta das informações sobre as variáveis investigáveis (independentes), foi desenvolvido um questionário estruturado a fim de obter dados sociodemográficos, variáveis comportamentais, fatores do trabalho e características de saúde com possíveis associações aos DORT. Esse questionário teve a validade de seu conteúdo analisada por uma equipe de sete doutores antes do início da pesquisa, obtendo-se um índice de validade de conteúdo de 0,98.

Deste modo, o questionário foi constituído por 19 variáveis independentes, separadas em quatro classes: 1) sociodemográficas (sexo, idade e escolaridade); 2) comportamentais (atividade física, tabagismo, tempo na postura sentada além do trabalho e uso do computador após o trabalho); 3) ocupacionais (horas extras, tempo diário sentado no trabalho e tempo de trabalho predominantemente na postura sentada – considerando ocupações anteriores, frequência do uso do computador, condições ergonômicas do posto de trabalho e índice de capacidade para o trabalho – ICT); e 4) saúde (uso de medicamento para dor/desconforto osteomuscular nos últimos 12 meses, circunferência da cintura, flexibilidade da cadeia muscular posterior, força muscular da porção inferior dos abdominais, encurtamento dos flexores do quadril e resistência dos músculos abdominais).

A variável dependente foi a prevalência de SO nos últimos 12 meses, investigada com o Questionário Nórdico de Sintomas Osteomusculares (QNSO), em sua versão geral, já validado no Brasil^13^. A interpretação desse desfecho foi realizada de forma numérica, preservando os relatos dos trabalhadores, que poderiam citar nenhum (zero) ou até nove SO.

Para a identificação relacionada à prática de atividade física foi utilizada a classificação da OMS, que recomenda para adultos 150 minutos de atividade física aeróbica de intensidade moderada por semana, ou pelo menos 75 minutos em intensidade vigorosa, sendo aceita também uma combinação equivalente de atividades de intensidade moderada e vigorosa^8^.

A avaliação das condições ergonômicas dos postos de trabalho informatizados foi realizada com o *checklist* de Couto, versão 2014^14^. Já para avaliar a capacidade laboral, foi utilizado o ICT em versão traduzida e adaptada para o Brasil^15^.

Optou-se, neste estudo, por utilizar a terminologia “circunferência da cintura” (CC), com base em estudo de revisão sistemática^16^, o ponto médio entre a última costela e a crista ilíaca como sítio anatômico de medida e pontos de corte segundo a International Diabetes Federation^17^ – o padrão mais utilizado internacionalmente e adotado pela Sociedade Brasileira de Cardiologia.

A força dos músculos abdominais inferiores foi identificada de acordo com o procedimento sugerido por Kendall et al.^18^ Já para avaliar o encurtamento dos músculos flexores do quadril, foi utilizado o teste de Thomas, conforme propõe Magee^19^. A flexibilidade dos músculos da região posterior do tronco e dos membros inferiores, denominada também de cadeia muscular posterior, foi medida pelo teste de sentar e alcançar com banco de Wells, por ser um método muito utilizado em pesquisa^20^.

A resistência ou *endurance* dos músculos abdominais foi avaliada com o teste do abdominal de um minuto, que consiste em realizar o número máximo de abdominais possível nesse intervalo de tempo. Os valores normativos para esse teste consideram o sexo e a idade da pessoa e classificam a resistência em excelente, acima da média, média, abaixo da média ou fraca^21^.

### Procedimentos de Coleta e Análise dos Dados

A coleta de dados foi realizada por uma equipe de cinco examinadores, que receberam um treinamento de 20 horas sobre aspectos relacionados às técnicas de entrevista, aos métodos e aos procedimentos dos testes e instrumentos, objetivando padronização e calibração. Foi criado um grupo em aplicativo de comunicação no celular para resolução de dúvidas imediatas, além de serem feitas reuniões presenciais quinzenais. As entrevistas e os testes físicos foram realizados no próprio local de trabalho, porém em sala privada – devido ao uso de colchonete e banco de Wells e para preservar a privacidade do participante –, com duração média de 35 minutos.

### Análises Estatísticas

A apresentação dos resultados foi realizada mediante uma abordagem descritiva e outra analítica. Na abordagem descritiva, apresentam-se a distribuição de frequência absoluta e relativa e medidas de tendência central para descrever as variáveis quantitativas. Quanto à abordagem analítica, foi obtido um intervalo com 95% de confiança (IC95%) para estimar a prevalência de SO, e foram feitas análises univariadas na comparação de todas as variáveis independentes com a dependente. Já na definição dos grupos de comparação segundo as categorias da variável ou na categorização de variáveis numéricas, para amostras grandes (n > 30) foram utilizados os testes paramétricos *t* de Student (para dois grupos independentes) e Anova (para três ou mais grupos independentes). Em todos os casos, intervalos com 95% de confiança foram obtidos para as médias. Para as variáveis numéricas, sem categorização, foi empregado o teste de correlação linear de Pearson.

Nas amostras pequenas e no caso de normalidade não satisfeita, verificada por meio do teste Shapiro-Wilk, foi empregado o teste não paramétrico Kruskal-Wallis (para três ou mais grupos independentes). A homocedasticidade entre grupos foi verificada pelo teste de Levene (paramétrico) ou pelo teste de Fligner-Killer (não paramétrico). Optou-se pela análise de regressão de Poisson com variância robusta, uma vez que é referência para análise de dados de contagem e pelo fato de o *odds ratio* ter a tendência de superestimar a razão de prevalência quando o desfecho é comum ou elevado^22^.

Utilizou-se um modelo no qual as variáveis independentes foram inseridas em blocos na seguinte ordem: dados sociodemográficos, comportamentais, ocupacionais e de saúde. Foram incluídas as variáveis com significância menor que 0,20 (p < 0,20) nas análises univariadas. A seleção das variáveis no modelo foi realizada com o critério *backward stepwise*, considerando-se também um valor igual a 0,20 para a estatística de Wald na manutenção das variáveis durante a análise ajustada nível a nível, a fim de controlar potenciais fatores de confusão. As medidas de efeito foram expressas em aumento relativo (AR) na média.

As análises finais foram consideradas em um nível de significância de 5% (α = 0,05). Os testes foram realizados nos programas estatísticos R versão 3.4.2 e *Statistical Package for the Social Sciences* (SPSS) versão 22.0. A pesquisa foi aprovada em 21 de novembro de 2017 pelo Comitê de Ética em Pesquisa, envolvendo Seres Humanos da Escola de Enfermagem de Ribeirão Preto da Universidade de São Paulo sob o protocolo CAAE 74543517.8.0000.5393, onde todos os participantes assinaram termo de consentimento livre e esclarecido.

## RESULTADOS

Participaram da pesquisa 451 trabalhadores, com idade média de 44,4 anos, sendo a maioria do sexo feminino (54,5%), com ensino superior ou pós-graduação (81,2%), não fumante (84,3%) e praticante de atividade física regularmente (53,9%). Permaneciam sentados em média 6,51 horas no trabalho e mais 3,12 horas durante o tempo de lazer/descanso. As demais características comportamentais, ocupacionais e de saúde encontram-se na Tabela 1, e as variáveis numéricas na Tabela 2. Não houve significância estatística em qualquer análise de correlação das variáveis numéricas com o número de SO nos últimos 12 meses relatado pelos trabalhadores (Tabela 2).

**Tabela 1:** Distribuição dos participantes segundo características sociodemográficas, comportamentais, ocupacionais e de saúde (n = 451) em municípios da região Sul do Brasil.

		Frequência (n)	Porcentagem (%)
Sexo	Feminino	246	54,5
	Masculino	205	45,5
Escolaridade	Ensino médio	62	13,7
	Ensino técnico	23	5,1
	Ensino superior	260	57,6
	Pós-graduação	106	23,6
Tabagismo	Fumante diário	17	3,8
	Fumante ocasional	9	2,0
	Ex-fumante	45	10,0
	Não fumante	380	84,3
Prática de atividade física	Sim	243	53,9
	Não	208	46,1
Uso do computador fora do trabalho	Sim	179	39,7
	Não	272	60,3
Hora extra	Sim	34	7,5
	Não	417	92,5
Frequência do uso do computador no trabalho	Menos de 50% do tempo	13	2,9
	Entre 50% e 70% do tempo	35	7,8
	Acima de 70% do tempo	403	89,4
ICT	Baixo	6	1,3
	Moderado	79	17,5
	Bom	204	45,2
	Ótimo	162	35,9
Uso de medicamento nos últimos 12 meses	Sim	244	54,1
	Não	207	45,9
Circunferência da cintura	Menor risco	202	44,8
	Risco aumentado	147	32,6
	Risco substancialmente aumentado	102	22,6
Flexibilidade (banco de Wells)	Excelente	41	9,1
	Acima da média	63	14,0
	Média	69	15,3
	Abaixo da média	81	18,0
	Ruim	197	43,7
Força muscular dos abdominais inferiores	Regular	170	37,7
	Boa	235	52,1
	Normal	46	10,2
Resistência dos abdominais	Excelente	94	20,8
	Acima da média	81	18,0
	Média	74	16,4
	Abaixo da média	66	14,6
	Fraca	136	30,2
Encurtamento dos flexores do quadril	Sim	80	17,7
	Não	371	82,3

ICT: índice de capacidade para o trabalho.

**Tabela 2:** Dados descritivos e análises de correlação das variáveis numéricas com a quantidade de sintomas osteomusculares nos últimos 12 meses (n = 451) em municípios da região Sul do Brasil.

	Idade (anos)	Tempo sentado fora do trabalho (horas/dia)	Tempo sentado no trabalho (horas/dia)	Tempo em trabalho sentado (em anos)	Média final do checklist de Couto (em %)
Dados descritivos: valor médio e desvio-padrão (DP)	44,4 (DP = 10,6)	3,12 (DP = 1,55)	6,51 (DP = 0,96)	20,29 (DP = 10,88)	88,9% (DP = 4,37)
Quantidade de SO nos últimos 12 meses					
Coeficiente de correlação^a^	0,083	0,082	0,033	0,079	0,068
Significância bilateral	0,077	0,082	0,489	0,093	0,152

SO: sintomas osteomusculares.

^a^ Teste de correlação de Pearson.

A prevalência estimada de SO nos últimos 12 meses foi de 90% (IC95% 87–93), com média de 3,34 sintomas por participante. Na análise dos SO em relação às variáveis sociodemográficas, observou-se maior frequência entre as mulheres, com média de 3,68 SO nos últimos 12 meses, enquanto os homens obtiveram uma média de 2,94, com p < 0,001. Em relação à escolaridade, aqueles com ensino técnico apresentaram a menor mediana de SO quando comparados aos participantes com outros níveis de escolaridade, com p = 0,027 (Tabela 3).

**Tabela 3:** Dados sociodemográficos e comportamentais em relação com os sintomas osteomusculares nos últimos 12 meses (n = 451) em municípios da região Sul do Brasil.

		Média	Desvio-padrão	IC95%	Mediana	Mínimo	Máximo	p
Sexo	Feminino	3,68	2,168	3,41–3,96	4,00	0	9	**< 0,001**
	Masculino	2,94	2,154	2,64–3,23	3,00	0	9	
Teste *t* de Student								
Escolaridade	Ensino médio	-	-	-	3,00	0	9	**0,027**
	Ensino técnico	-	-	-	2,00	0	6	
	Ensino superior	-	-	-	3,00	0	9	
	Pós-graduação	-	-	-	3,00	0	9	
Teste Kruskal-Wallis								
Tabagismo	Fumante diário	-	-	-	5,00	0	8	0,397
	Fumante ocasional	-	-	-	4,00	1	6	
	Ex-fumante	-	-	-	3,00	0	7	
	Não fumante	-	-	-	3,00	0	9	
Teste Kruskal-Wallis								
Prática de atividade física	Sim	3,11	2,078	2,84–3,37	3,00	0	9	**0,014**
	Não	3,62	2,289	3,31–3,93	4,00	0	9	
Teste *t* de Student								
Uso do computador fora do trabalho	Sim	3,23	2,173	2,91–3,56	3,00	0	9	0,392
	Não	3,42	2,204	3,15–3,68	3,00	0	9	
Teste *t* de Student								

IC95%: intervalo com 95% de confiança.

Valores com significância estatística estão apresentados em negrito.

Quanto às variáveis comportamentais, os inativos fisicamente apresentaram maior média de SO (*x¯* = 3,62) que os que praticavam atividade física (*x¯* = 3,11), com p = 0,014. Já na análise do hábito de fumar e do uso do computador fora do trabalho, não foram observadas diferenças significativas entre os grupos (Tabela 3).

Quanto à relação dos SO com as variáveis ocupacionais, ficou demonstrado que os trabalhadores com ICT baixo também tiveram mais SO, com mediana de 5,5, enquanto os que obtiveram ótimo ICT apresentaram mediana de 2, com p < 0,001 (Tabela 4).

**Tabela 4:** Dados ocupacionais e de saúde em relação com os sintomas osteomusculares nos últimos 12 meses (n = 451) em municípios da região Sul do Brasil.

		Média	Desvio-padrão	IC95%	Mediana	Mínimo	Máximo	p
Hora extra	Sim	3,15	1,893	2,49–3,81	3,00	0	7	0,587
	Não	3,36	2,214	3,15–3,57	3,00	0	9	
Teste *t* de Student								
Frequência do uso do computador no trabalho	Menos de 50% do tempo	-	-	-	2,00	1	7	0,614
	Entre 50% e 70% do tempo	-	-	-	3,00	0	8	
	Acima de 70% do tempo	-	-	-	3,00	0	9	
Teste Kruskal-Wallis								
ICT	Baixo	-	-	-	5,50	2	9	**< 0,001**
	Moderado	-	-	-	5,00	0	9	
	Bom	-	-	-	3,50	0	9	
	Ótimo	-	-	-	2,00	0	8	
Teste Kruskal-Wallis								
Uso de medicamento nos últimos 12 meses	Sim	4,10	2,031	3,85–4,36	4,00	0	9	**< 0,001**
	Não	2,45	2,033	2,17–2,73	2,00	0	9	
Teste *t* de Student								
Circunferência da cintura	Menor risco	3,08	2,098	2,79–3,37	3,00	0	9	**< 0,001**
	Risco aumentado	3,12	2,225	2,75–3,48	3,00	0	9	
	RSA	4,20	2,125	3,78–4,61	4,00	0	9	
Teste Anova								
Flexibilidade (banco de Wells)	Excelente	-	-	-	3,00	0	7	0,602
	Acima da média	-	-	-	3,00	0	8	
	Média	-	-	-	3,00	0	9	
	Abaixo da média	-	-	-	3,00	0	9	
	Ruim	-	-	-	3,00	0	9	
Teste Kruskal-Wallis								
Força muscular dos abdominais inferiores	Regular	3,61	2,182	3,28–3,94	3,50	0	9	0,134
	Boa	3,17	2,248	2,88–3,45	3,00	0	9	
	Normal	3,28	1,846	2,73–3,83	3,00	0	8	
Teste Anova								
Resistência dos abdominais	Excelente	2,85	2,190	2,40–3,30	2,50	0	9	0,072
	Acima da média	3,40	2,017	2,95–3,84	3,00	0	9	
	Média	3,46	2,121	2,97–3,95	3,00	0	8	
	Abaixo da média	3,17	2,351	2,59–3,74	3,00	0	9	
	Fraca	3,68	2,211	3,30–4,05	3,50	0	9	
Teste Anova								
Encurtamento dos flexores do quadril	Sim	3,63	2,420	3,09–4,16	3,00	0	9	0,245
	Não	3,28	2,137	3,06–3,50	3,00	0	9	
Teste *t* de Student								

SO: sintomas osteomusculares; ICT: índice de capacidade para o trabalho; RSA: risco substancialmente aumentado; IC95%: intervalo com 95% de confiança.

Valores com significância estatística estão apresentados em negrito.

As análises univariadas das variáveis de saúde em relação à presença de SO nos últimos 12 meses mostraram que as pessoas que utilizaram medicamentos para dor no último ano obtiveram maior média de SO (*x¯* = 4,10) do que as que não utilizaram (*x¯* = 2,45), com p < 0,001 (Tabela 4).

Os trabalhadores classificados, por meio da medida da circunferência da cintura, na categoria de risco substancialmente aumentado para doenças metabólicas e outras complicações tiveram média de SO mais elevada (4,20) do que os que estavam em menor risco (3,08), com p < 0,001. Na análise da flexibilidade, não houve diferença estatisticamente significativa entre os grupos (p = 0,602), e todas as categorias de classificações obtiveram mediana igual a 3 (Tabela 4).

Avaliando a relação com a força muscular dos abdominais inferiores, aqueles com força regular registraram uma média de SO de 3,61 e IC95% 3,28–3,94. Já no estudo da resistência dos músculos abdominais, avaliada no teste de um minuto, não foi constatada diferença significativa entre os grupos, sendo a média dos trabalhadores com fraca resistência muscular de 3,68 SO. Com valor muito similar, aqueles com encurtamento dos flexores do quadril obtiveram média de SO de *x¯* = 3,63 e IC95% 3,09–4,16, sem diferença significativa entre os grupos (Tabela 4).

A partir dos resultados das análises univariadas, foram selecionadas para o modelo de regressão doze variáveis: idade; sexo; escolaridade; prática de atividade física; tempo sentado fora do trabalho; tempo em trabalhos predominantemente na postura sentada; condições ergonômicas do posto de trabalho; ICT; uso de medicamentos nos últimos 12 meses; circunferência da cintura; força muscular dos abdominais inferiores; e resistência dos abdominais.

Os resultados da análise de regressão múltipla, após todas as etapas por blocos (variáveis sociodemográficas, comportamentais, ocupacionais e de saúde), mostraram cinco fatores associados significativamente à média de SO. A Tabela 5 apresenta todas as variáveis que permaneceram no modelo até o final da análise e seus respectivos valores estimados de AR na média, IC95% e significância estatística (p-valor).

**Tabela 5:** Modelo de regressão de Poisson com variância robusta em (n = 451) municípios da região Sul do Brasil.

Parâmetros	Estimativa	Erro-padrão	z-valor	p-valor	AR	IC95%	
(*Intercept*)	0,6763	0,1090	6,2061	0,0000	1,9667		
Sexo feminino	0,1376	0,0626	2,1967	**0,0280**	1,1475	1,0149	1,2974
Escolaridade: ensino técnico	-0,4535	0,1788	-2,5361	**0,0112**	0,6354	0,4475	0,9021
Escolaridade: ensino superior	-0,1134	0,0851	-1,3323	0,1828	0,8928	0,7556	1,0549
Escolaridade: pós-graduação	-0,0573	0,0999	-0,5739	0,5660	0,9443	0,7764	1,1485
ICT baixo	0,6933	0,2050	3,3817	**0,0007**	2,0002	1,3384	2,9894
ICT moderado	0,4950	0,0868	5,7035	**0,0000**	1,6406	1,3839	1,9448
Uso de medicamentos nos últimos 12 meses	0,3924	0,0643	6,1008	**0,0000**	1,4806	1,3052	1,6795
CC em risco aumentado	-0,0421	0,0702	-0,5996	0,5488	0,9588	0,8355	1,1002
CC em risco substancialmente aumentado	0,1449	0,0738	1,9630	**0,0496**	1,1559	1,0002	1,3358

AR: aumento relativo na média; IC95%: intervalo com 95% de confiança; ICT: índice de capacidade para o trabalho; CC: circunferência da cintura.

Valores com significância estatística estão apresentados em negrito.

As mulheres tiveram um AR na média de SO de 14,75% (p = 0,0280) em comparação com os homens. Na análise da escolaridade, os trabalhadores com ensino técnico apresentaram redução de 36,46% (p = 0,0112) na média de SO em relação àqueles com ensino médio, demostrando ser este um fator de proteção (Tabela 5).

Os trabalhadores com ICT baixo e moderado apresentaram AR de 100,02% (p = 0,0007) e 64,06% (p < 0,0001) na média do número de sintomas, respectivamente, em relação aos com ICT ótimo. Já os participantes que fizeram uso de medicamentos para dores osteomusculares nos últimos 12 meses tiveram AR na média de 48,06% (p < 0,0001) quando comparados aos que não utilizaram medicamentos (Tabela 5).

Por fim, na medida da circunferência da cintura, os participantes com risco substancialmente aumentado para síndrome metabólica e outras complicações apresentaram AR na média de SO de 15,59% (p = 0,0496) com relação aos trabalhadores com menor risco (Tabela 5).

## DISCUSSÃO

A estimativa da prevalência de SO nos últimos 12 meses foi elevada (90%). Alguns estudos constataram alta prevalência de distúrbios osteomusculares entre indivíduos que trabalham predominantemente sentados, como os trabalhadores de escritórios (88,4%)^10^ e informática (76%)^11^.

As dores foram significativamente mais frequentes nas mulheres quando comparadas aos homens, com AR de 14,75% na média de sintomas. Esse dado é regularmente relatado por autores que estudam SO em trabalhadores. No estudo de Scopel et al.^12^, os homens tiveram menor prevalência de casos sugestivos de DORT, com razão de prevalência estimada de 0,62 (IC95% 0,47–0,81).

Entre os trabalhadores de escritório que utilizam computador, as mulheres apresentaram mais SO em todas as regiões anatômicas avaliadas, e na análise de associação multivariada obtiveram uma razão de chance ou *odds ratio* igual a 2,4 (p = 0,03) para a região da cervical e igual a 2,8 (p = 0,01) para membros superiores (braço, cotovelo, antebraço, punho ou mão)^11^. Algumas possíveis explicações para esses achados seriam a menor massa muscular, em termos absolutos e relativos, na composição física da mulher^19^; alterações no sistema hormonal; e a dupla jornada de trabalho, já que muitas vezes ela é a responsável pelos cuidados familiares.

Na análise da escolaridade, aqueles que tinham curso técnico apresentaram menos sintomas, servindo isso como um fator de proteção. Parece não haver padrão estabelecido quanto a essa variável. Algumas pesquisas demostraram não haver associação da escolaridade com SO^23,24^, enquanto outras encontraram uma associação de SO com menor grau de estudos^12,25^.

Os trabalhadores classificados com ICT baixo apresentaram um AR na média de SO significante em relação aos com ICT ótimo. Iunes et al.^26^ evidenciaram associação entre o ICT e a presença de sintomas em todas as regiões descritas no QNSO.

Walsh et al.^27^ observaram que, dentre os trabalhadores com ICT pobre/baixo, 87% tinham intensidade da dor entre 7 e 10, enquanto 73% dos com ICT excelente pontuaram de 0 a 2. No estudo de Martinez e Latorre^28^, todas as dimensões da saúde analisadas, inclusive a avaliação da dor, foram associadas à capacidade para o trabalho, sendo esta tão melhor quanto maior for a qualidade da saúde.

Essa relação parece ocorrer nos dois sentidos, ou seja, o trabalhador que possui um número elevado de SO tende a ter um baixo ICT, e aquele que possui um alto ICT tem menor tendência de apresentar SO relacionados ao trabalho. Manter a capacidade para o trabalho é um desafio para os serviços de saúde e o ICT , juntamente com outras avaliações, fornecem aos profissionais e gestores ferramentas e dados essenciais para acompanhar a saúde do trabalhador, permitindo medidas de prevenção e promoção da saúde no ambiente laboral.

Analisando as variáveis de saúde, houve diferença estatisticamente significativa entre os que utilizaram medicamento para dores osteomusculares no último ano e os que não utilizaram. Na análise de regressão observou-se associação significativa, expressando um AR de 48,06% na média de sintomas. Esse dado pode ser facilmente compreendido, pois a pessoa com dor é mais propensa a utilizar medicamentos. No estudo de Souza et al.^29^, 64,51% dos trabalhadores de um hospital público fizeram uso de medicamento para dor na semana anterior à pesquisa.

A associação da circunferência da cintura dos trabalhadores classificados em risco substancialmente aumentado (indicador de obesidade central ou abdominal) com a média de SO manteve-se estatisticamente significativa na análise univariada até o modelo final da regressão de Poisson, com AR de 15,59%. Magnago et al.^25^ constataram que quase metade dos trabalhadores de enfermagem de um grande hospital universitário do Rio Grande do Sul estava acima do peso ideal, e esse dado foi associado significativamente aos relatos de dor em articulações e coluna lombar.

Para uma possível compreensão dessa associação, vale citar o efeito da sobrecarga nas articulações que ocorre nas pessoas com sobrepeso e obesidade. O estresse e a pressão excessiva causam desgastes nas estruturas e nos tecidos envolvidos (cartilagens, ligamentos, tendões, músculos, entre outros), predispondo à degeneração e à dor^30^.

Alguns pontos relevantes e limitações desta pesquisa merecem destaque, a começar pela apresentação e análise dos dados sobre SO na perspectiva numérica, que considera a influência de cada sintoma relatado e mantém a coerência com o modelo matemático da regressão de Poisson. Outra observação é a apresentação clínica, que pode se mostrar mais relevante e pertinente ao profissional da saúde, visto que em intervenções preventivas e/ou reabilitativas é importante identificar os fatores associados capazes de aumentar o número de SO nos trabalhadores, sem se restringir a cada região corporal do QNSO.

Além das limitações típicas de estudos transversais, como a impossibilidade de estabelecer causalidade, vale citar a questão do autorrelato do tempo sentado, embora a literatura já demonstre certa confiabilidade. Não se observou viés do voluntário sadio, haja vista a alta prevalência de SO, porém deve-se considerar o viés do trabalhador saudável, tendo em conta a exclusão dos trabalhadores afastados por motivos de doença.

Este estudo contribui para o avanço do conhecimento sobre a saúde dos trabalhadores que, independentemente de praticarem atividade física, podem ser classificados num comportamento/estilo de vida sedentário, pois a natureza de sua ocupação profissional exige a postura sentada por longos períodos, diariamente^9^, de modo que ficam submetidos a esse fator de risco, considerado constructo distinto de inatividade física e de crescente interesse na saúde pública.

Portanto, as variáveis sexo feminino, ICT baixo e moderado, uso de medicamentos nos últimos 12 meses e circunferência da cintura acima do recomendável foram estatisticamente associadas ao aumento de SO, sendo a variável ICT a de maior poder de associação. Constatou-se ainda alta prevalência de SO nesses trabalhadores. Isto posto, intervenções para diminuir os SO irão repercutir positivamente na capacidade para o trabalho, o que, por sua vez, tende a diminuir a presença de sintomas.

Assim sendo, uma medida imediata a se adotar é a reabilitação dos trabalhadores com SO. Sabe-se que sintomas iniciais, se negligenciados, podem evoluir para distúrbios incapacitantes, tornando necessário o recurso a afastamentos e auxílios governamentais – o que aumenta um grande problema de saúde pública no Brasil, com repercussões nas dimensões sociais e econômicas.

Além disso, algumas ações peculiares são propostas: adaptações físicas no local de trabalho e ajustes/mudanças na organização das atividades para que os trabalhadores possam ficar em pé por alguns minutos a cada hora e tenham maior gasto energético; orientações e esclarecimentos, com profissionais da saúde especializados, sobre comportamentos de risco e hábitos saudáveis no ambiente laboral; e realização de exercícios específicos para o sistema osteomuscular, que estimulem a circulação sanguínea e desenvolvam características físicas individuais comprovadamente protetoras para quem fica sentado por longos períodos.
